# Approach to eyebrow ptosis through the modified technique of Castanãres

**DOI:** 10.4103/0970-0358.53013

**Published:** 2009

**Authors:** Pires Viana Giovanni, Pires Viana Giovanni André

**Affiliations:** Member of Brazilian Plastic Surgery Society, Member of Brazilian College of Surgeons; 1 Member of International College of Surgeons

**Keywords:** Arched eyebrow, Eyebrow position, Eyebrow ptosis, Surgery for eyebrow ptosis

## Abstract

One of the first signs of facial ageing appears in the forehead, with the descent of the lateral part of the brow. This is a troubling condition for all patients and elevation of the lateral part of the brow becomes a more and more frequent demand. The authors present their experience in 350 consecutive cases of direct eyebrow lift (“butterfly wing” incision) alone or in combination with rhytidectomy and/or blepharoplasty. The majority of the patients were female (90%). The age ranged from 43 to 85 years. Eighty per cent of the cases were performed simultaneously with rhytidectomy and blepharoplasty, 16% were performed in association with blepharoplasty and eyebrow lift alone represented 4%. The most common complication was epidermal cyst (3.4%) and suture dehiscence (2%). The “butterfly wing” incision provides a useful alternative to correction of eyebrow ptosis. Indication for surgery is dependent more on the ageing signs than on the patient's chronological age. The final results are consistently very gratifying to the patient and to the plastic surgeon alike.

## INTRODUCTION

Fascination with beauty as well as with the orbits and their surrounding tissues dates back to early human civilization. The lid–eyebrow region forms the emotional and expressive centre of the human face.[1] A high eyebrow positioned above the orbital rim and small eyebrows with the eyebrow arch positioned in the middle were preferred for many decades.[1,2] Ageing causes decreased skin elasticity, which allows soft tissue to descend with gravity. Ptotic eyebrows can be hereditary, appearing early in life. The resultant brow ptosis manifests in lateral hooding of the eyelid and has implications for functional visual field obstruction as well as periocular cosmetic surgery.

Surgical elevation of the forehead and brow for aesthetic improvement of the upper third of the ageing face has been performed for nearly 100 years.[3] Current methods of eyebrow lifting include direct eyebrow lifts, midforehead lifts, coronal brow lifts and endoscopic brow lifts.[1,3,4–13] More recently, nonendoscopic, limited-incision approaches to correct the descent of the lateral eyebrow alone have been reported by several authors.[4,6,10,14–16]

The aim of this paper is to analyze the treatment of ptosis of the tail of the eyebrow by the modified technique of Castanãres in 350 consecutive patients who underwent direct eyebrow lift alone or in combination with rhytidectomy and/or blepharoplasty at a private clinic between January 1996 and December 2007.

## MATERIAL AND METHODS

Between January 1996 and December 2007, 350 consecutive patients underwent a direct eyebrow lift alone or in combination with rhytidectomy and/or blepharoplasty. Each patient was evaluated for effective brow elevation, complications and overall satisfaction.

### Surgical procedure

**Figure 1:**
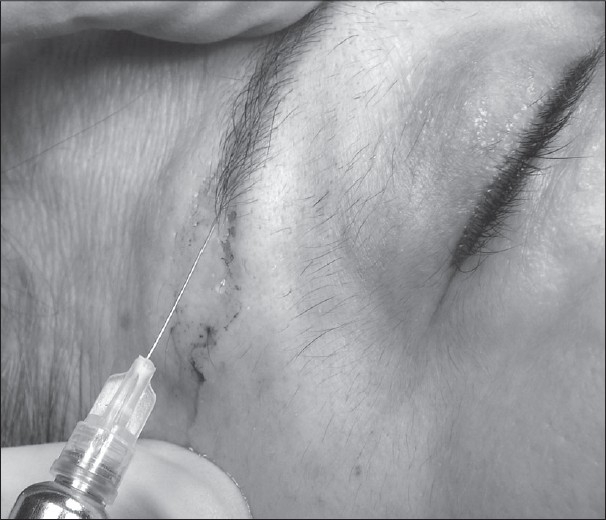
Anaesthetic injection into the “butterfly wing” incision

All patients were operated upon with monitored local anaesthesia. The local anaesthesic (2% lidocaine with 1:200,000 epinephrine) was injected into the “butterfly wing” incision [Figure 1]. This drawing was proposed initially by Vinãs[5] and popularized by Castanãres.[4]

The amount of skin resection depends on the elevation desired and the amount calculated ahead of time to be excessive. Viñas has suggested a simple but accurate method to determine the amount of skin resection: pinching the skin with the thumb and index finger above the lateral end of the brow with the patient in the erect position and watching for the desired effect.[5]

**Figure 2:**
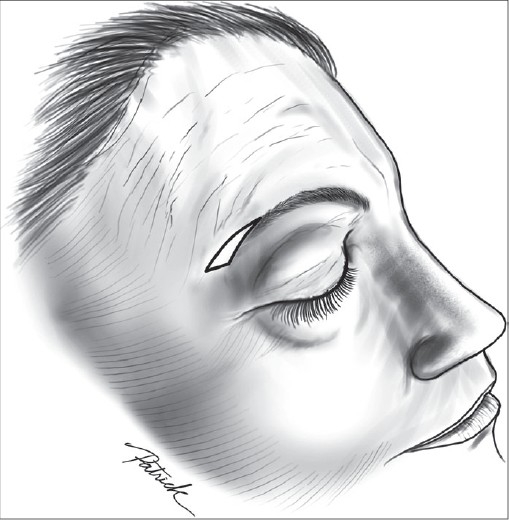
Schematic drawing: As described by Castañares, the widest part of the ellipse is marked above the tail of the brow. The lateral extension is then carried out to complete the ellipse beyond the tail of the brow in an upward and lateral direction. The lateral apex of the ellipse falls slightly above the level of the centre of the brow in an imaginary horizontal line. If the skin resection were in the shape of a crescent, arching it exactly over and parallel to the brow itself

**Figure 3:**
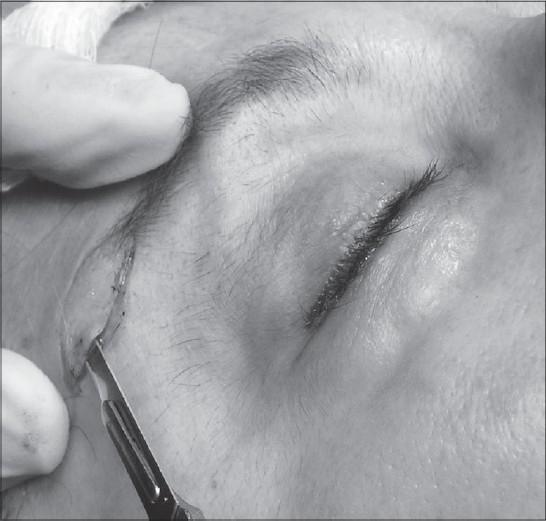
Underming superficially

After drawing the incision [Figure 2] and injection of the local anaesthesic, the authors undermine superficially (epidermis dissection), like Schwartzman's maneuver in breast reduction surgery [Figure 3]. There is no undermining underneath this level. Then, the epidermis is removed and the closure is carried out in the conventional manner. A pressure dressing is applied to the incision for 5 days.

## RESULTS

700 eyebrow elevations were performed in 350 consecutive patients whose ages ranged from 43 to 85 years. 90% of the patients were female. 90% of the cases were performed simultaneously with rhytidectomy and blepharoplasty, 16% were performed in association with blepharoplasty and eyebrow lift alone represented 4% of the cases. The procedure was completed on average in 30 min (20.45 min).

The overall rate of complications was low. The most common complication was epidermal cyst (3.4%) and suture dehiscence due to local trauma (2%). Reoperation was performed in the unsatisfied patients (2%). There were no hypertrophic or keloid scars, no infection, no lagophthalmos and no post operative haematoma.

**Figure 4:**
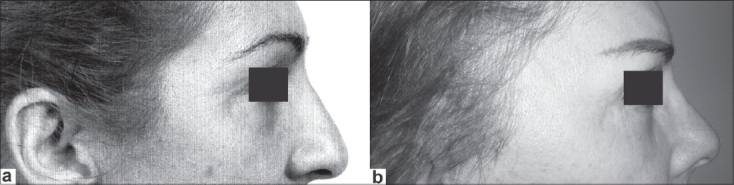
Female 58 years old: (a) preoperative view (1997) and (b) postoperative view (10 years)

**Figure 5:**
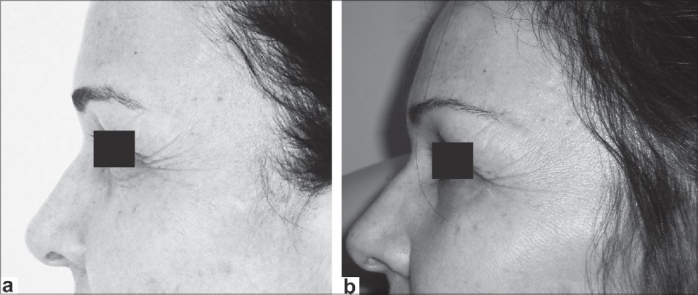
Female 55 years old: (a) preoperative view and (b) postoperative view (20 days)

**Figure 6:**
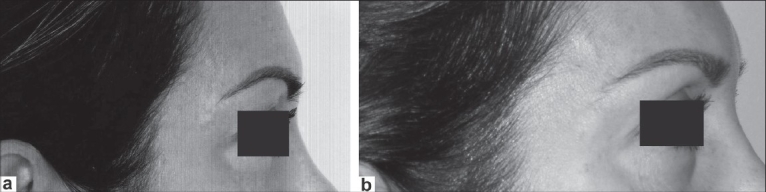
Female 48 years old: (a) preoperative view and (b) postoperative view (3 years)

**Figure 7:**
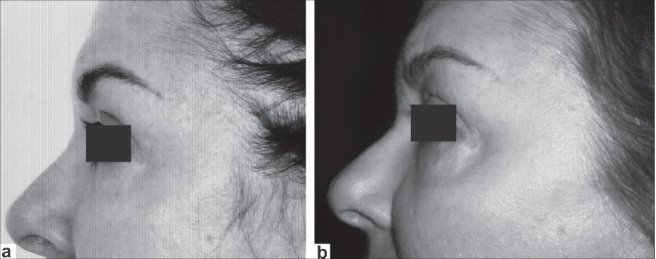
Female 61 years old (a) preoperative view and (b) postoperative view (1 year)

The follow-up in this series was from 10 months to 11 years. Good brow elevation that lasted through the period of follow-up was uniformly demonstrated [Figures 4–7].

## DISCUSSION

The modern concept of “ideal” brow position was described by Westmore in 1974.[1,15] However, there is a great diversity among individuals with respect to eyebrow position and shape and the notion of an “ideal” eyebrow has changed quite significantly over the past several decades, as demonstrated by Feser *et al*.[1] According to these authors there are atleast three ideals for a beautiful eyebrow; this being determined by the patients' age for the young tend to prefer eyebrows in a lower position. This way, it seems plausible to assume that the trend currently appears to be moving away from arched eyebrows toward lower-positioned eyebrows with maximum height in the lateral third.[1]

Knize discussed several mechanisms contributing to brow ptosis, including depression of the medial eyebrow from overaction of the brow depressors and descent of the lateral eyebrow from unopposed lateral orbicularis oculi contraction.[16] To recreate the aesthetically pleasing brow, several surgical procedures have been published over the past century.[3–8,10–17] Many different surgical techniques and types of incisions for raising the brow have been characterized, including the classical approach correction through a transcoronal incision,[4–8,13,18–20] transpalpebral brow lift, endoscopic brow liftand the use of botulinum toxin.More recently, nonendoscopic, limited-incision approaches to correct the descent of the lateral eyebrow alone have been reported by several authors.[14–16] Sometimes a combination with brow lift and blepharoplasty is necessary to achieve the desired results.[13,21] Performing upper blepharoplasty in conjunction with brow lift is not a problem if care is given to proper preoperative analysis, quantification and marking.[13]

In this study, the authors present their experience with “butterfly wing” incision based on Viñas' study.[5] Indication for surgery is more dependent on the ageing signs than on the patient's chronological age. The author's use this approach to correct the lateral end of the brow (tail), mostly in patients with hairless eyebrow or in patients wearing makeup to disguise the brow ptosis. Nevertheless, this approach would be carried out in all patients because they have been warned ahead of time of the scar extension. It is also important to stress to the patient that the scar would be visible for a period of time but it may be concealed temporrarily with cosmetics.

Viñas[5] and Castañares[4] correct the eyebrow ptosis by resection of a roughly elliptical section of the forehead skin immediately above and lateral to the eyebrow. Through this approach the forehead is undermined to the hairline, severing all epicranial fibres, the corrugator supercilii and the procerus.[4] The authors' resection is performed superficially, like Schwartzman's maneuver in breast reduction surgery, without undermining underneath the epidermis level. In this manner, the authors believe that this approach reduces the prevalence of unaesthetic scar formation by avoiding injury to the dermis.

In this series, the most common complication was epidermal cyst (3.4%), suture dehiscence due to local trauma (2%) and reoperation of unsatisfied patients (2%). Reoperation was performed in seven patients with minimal recurrence of brow ptosis due to previous inadequate resection. Viñas *et al*.[5] revealed an extremely low complication rate (0.4%) of haematoma formation, permanent alopecia and “nerve damage”. They also reported a 2.4% dissatisfaction rate, which is comparable with the numbers presented in this series and other studies in literature.[3,5,7–9,13,15,16] According to Viñas,[5] the advantage of this approach is its simplicity, it is well tolerated as an outpatient procedure under local anaesthesia and that most patients can resume their activities the following day. The authors agree with Viñas in that the other advantage is that it carries a direct one-to-one correction of brow ptosis. The disadvantage of this procedure is the scars on the forehead. To quote Viñas:.The scars usually become inconspicuous with time. Also because of their location, they can be easily disguised (if necessary) with a couple of strokes of an eyebrow pencil”.[5] If these factors are properly explained beforehand and with the visible improvement, these patients accept the temporary inconveniences with conformity and understanding.

The “butterfly wing” incision provides a useful alternative in situations in which the surgeon may not be familiar with endoscopic techniques, does not have access to endoscopic equipment or when frontalis modification is not required. Although this operation yields sustained improvements, it does not remove all underlying factors involved in brow ageing. The effects of gravitational forces continue and may result in the need for future enhancements. The final results of this operation are consistently very gratifying to the patient and to the plastic surgeon alike.

## References

[1] Feser DF, Gründl M, Eisenmann-Klein M, Prantl L (2007). Attractiveness of eyebrow position and shape in females depends on the age of the beholder. Aesth Plast Surg.

[2] Gunter JP, Antrobus SD (1997). Aesthetic analysis of the eyebrows. Plast Reconstr Surg.

[3] Paul MD (2001). The evolution of the brow lift in aesthetic plastic surgery. Plast Reconstr Surg.

[4] Castañares S (1964). Forehead wrinkles, glabellar frown and ptosis of the eyebrows. Plast Reconstr Surg.

[5] Viñas JC, Caviglia C, Cortiñas JL (1976). Forehead rhytidoplasty and brow lifting. Plast Reconstr Surg.

[6] Herzog Neto G, Sebastiá R, Viana GAP (2005). Brow ptosis: Transpalpebral approach. Rev Soc Bras Cir Plast.

[7] Pitanguy I (1981). Indications for and treatment of frontal and glabellar wrinkles in an analysis of 3,404 consecutive cases of rhytidectomy. Plast Reconstr Surg.

[8] Berkowitz RL, Jacobs DI, Gorman PJ (2005). Brow fixation with the endotine forehead device in endoscopic brow lift. Plast Reconstr Surg.

[9] Elkwood A, Matarasso A, Rankin M, Elkowitz M, Godek CP (2001). National plastic surgery survey: Brow lifting techniques and complications. Plast Reconstr Surg.

[10] Lassus C (1999). Elevation of the lateral brow without the help of an endoscope. Aesth Plast Surg.

[11] Karabulut AB, Tümerderm B (2001). Forehead lift: A combined approach using subperiostal and subgaleal dissection planes. Aesth Plast Surg.

[12] Troilius C (2004). Subperiostal brow lifts without fixation. Plast Reconstr Surg.

[13] Friedland JA, Jacobsen WM, TerKonda S (1996). Safety and efficacy of combined upper blepharoplasty and open coronal browlift: A consecutive series of 600 patients. Aesth Plast Surg.

[14] Strauch B, Baum T (2002). Connection of lateral brow ptosis: A nonendoscopic subgaleal approach. Plast Reconstr Surg.

[15] Kikkawa DO, Miller SR, Batra MK, Lee AC (2000). Small incision nonendoscopic browlift. Ophthalmic Plast Reconstr Surg.

[16] Tabatabai N, Spinelli HM (2007). Limited incision nonendoscopic browlift. Plast Reconstr Surg.

[17] Knize DM (1996). An anatomically based study of the mechanism of eyebrow ptosis. Plast Reconstr Surg.

[18] Paul MD (1989). The surgical management of upper eyelid hooding. Aesth Plast Surg.

[19] Ramirez OM (1995). Endoscopically assisted biplanar forehead lift. Plast Reconstr Surg.

[20] Fagien S (1999). Botox for the treatment of dynamic and hyperkinetic facial lins and furrows: Adjunctive use in facial aesthetic surgery. Plast Reconstr Surg.

[21] Rohrich RJ, Coberly DM, Fagien S, Stuzin JM (2004). Current concepts in aesthetic upper blepharoplasty. Plast Reconstr Surg.

